# PTH1 Receptor Is Involved in Mediating Cellular Response to Long-Chain Polyunsaturated Fatty Acids

**DOI:** 10.1371/journal.pone.0052583

**Published:** 2012-12-27

**Authors:** Jose Candelario, Hesam Tavakoli, Mirianas Chachisvilis

**Affiliations:** La Jolla Bioengineering Institute, San Diego, California, United States of America; Westmead Millennium Institute, University of Sydney, Australia

## Abstract

The molecular pathways by which long chain polyunsaturated fatty acids (LCPUFA) influence skeletal health remain elusive. Both LCPUFA and parathyroid hormone type 1 receptor (PTH1R) are known to be involved in bone metabolism while any direct link between the two is yet to be established. Here we report that LCPUFA are capable of direct, PTH1R dependent activation of extracellular ligand-regulated kinases (ERK). From a wide range of fatty acids studied, varying in chain length, saturation, and position of double bonds, eicosapentaenoic (EPA) and docosahexaenoic fatty acids (DHA) caused the highest ERK phosphorylation. Moreover, EPA potentiated the effect of parathyroid hormone (PTH(1–34)) in a superagonistic manner. EPA or DHA dependent ERK phosphorylation was inhibited by the PTH1R antagonist and by knockdown of PTH1R. Inhibition of PTH1R downstream signaling molecules, protein kinases A (PKA) and C (PKC), reduced EPA and DHA dependent ERK phosphorylation indicating that fatty acids predominantly activate G-protein pathway and not the β-arrestin pathway. Using picosecond time-resolved fluorescence microscopy and a genetically engineered PTH1R sensor (PTH-CC), we detected conformational responses to EPA similar to those caused by PTH(1–34). PTH1R antagonist blocked the EPA induced conformational response of the PTH-CC. Competitive binding studies using fluorescence anisotropy technique showed that EPA and DHA competitively bind to and alter the affinity of PTH1 receptor to PTH(1–34) leading to a superagonistic response. Finally, we showed that EPA stimulates protein kinase B (Akt) phosphorylation in a PTH1R-dependent manner and affects the osteoblast survival pathway, by inhibiting glucocorticoid-induced cell death. Our findings demonstrate for the first time that LCPUFAs, EPA and DHA, can activate PTH1R receptor at nanomolar concentrations and consequently provide a putative molecular mechanism for the action of fatty acids in bone.

## Introduction

As age increases, the risk of bone loss and fracture increases [1]. With the growing increase in average life expectancy, the need to develop new strategies to prevent osteoporosis and fragility fractures is greater than ever [2]. It is believed that dietary modifications and physical activity may be considered the primary targets to minimize bone loss and fragility [3].

Parathyroid hormone (PTH) has been shown to play an important role in bone homeostasis [4]. Parathyroid hormone related protein (PTH-rP) is a vital developmental morphogen [5], [6]. PTH and PTH-rP both bind to and activate PTH/PTH-rP Receptor (PTH1R), a G-protein coupled receptor with seven transmembrane domains which is highly expressed in bone and kidney [7]. PTH1R stimulates multiple signaling cascades including the G_s_-cAMP-PKA [8], G_q_/11-PLC-PKC [9], and mitogen-activated protein kinases (MAPKs) leading to various biological effects including anabolic and catabolic actions in bone [10]. PTH has also been reported to stimulate phosphorylation of Akt [11], a critical regulator of osteoblast differentiation [12] and survival [13].

It is well documented that lipids play an important role in skeletal biology and bone health [14]. LCPUFA, best known for their cardio-protective role, can regulate bone metabolism [15], [16] and may potentially play a role in the prevention of osteoporosis. There are several biological pathways whereby polyunsaturated fatty acids may regulate bone metabolism. Fish oil, which contains large amounts of ω-3 fatty acids, is suggested to modulate a number of pro-inflammatory cytokines, increase production of insulin-like growth factor-1 (IGF-1), and improve calcium accretion in bone [17]. Consequently, it has been proposed that ω-3 fatty acids could prevent age-related bone loss by inhibiting osteoclastogenesis while improving osteoblast differentiation and function [18]. The effect of ω-3 fatty acids on the skeleton seems to be further dependent on the two main ω-3 fatty acids: EPA and DHA [19]. Long chain ω-3 fatty acids cause increased bone formation in chicks and rats suggesting a stimulatory effect on osteoblast activity [20].

In human studies, it has been shown that consuming EPA improved bone quality in elderly female subjects [21]. Consumption of ω-3 fatty acids was also associated with reduced incidence and severity of inflammatory bone/joint diseases in humans [22]. There is evidence of the potential of EPA to counteract bone loss associated with spaceflight; higher consumption of fish (ω-3) was associated with reduced loss of bone mineral density (BMD) after flight [23]. BMD of the total body showed a significant negative correlation with serum concentrations of oleic acids and monounsaturated fatty acids and significant correlations with DHA and ω-3 fatty acids [24]. A higher ratio of ω-6 to ω-3 fatty acids is associated with lower BMD at the hip in both sexes suggesting the relative amounts of dietary PUFA may play a vital role in preserving skeletal integrity in older age [25].

Studies on LCPUFA and CLA (conjugated linoleic acid) in laboratory animals suggest that dietary intakes of different PUFAs and CLA can affect bone remodeling through biosynthesis of prostaglandins and insulin-like growth factors [14]. Dietary supplementation with ω-3 polyunsaturated rich oils has been linked to increased calcium balance and bone formation rate during growth [26] as well as improved maintenance of bone mass post-ovariectomy [27]. Both DHA and total ω-3 PUFA strongly correlate with bone mineral content (BMC) in the femur of growing rats [28]. Reduced bone mineral loss was observed in ovariectomized rats supplemented with EPA [20].

The venous blood concentration of fatty acids is known to vary from 250 µM to 3 mM depending on the nutritional state [29]; most of these fatty acids are bound to serum albumin while a small percent is unbound in the plasma. The amounts incorporated into plasma membranes (PM) of cells can be enhanced by up to 10 times through dietary supplements enriched in LC-PUFA [30]–[32]. Further examination of blood fatty acid composition reported that roughly 5.3% of total serum fatty acids are LC-PUFA (2.4% docosahexaenoic (DHA), 1.8% eicosapentaenoic (EPA), 1% docosapentaenoic (DPA)) [33]; similar fatty acid concentration/composition has been reported in mice [34], [35]. Albumin concentration in interstitial fluid [36], [37] is at ∼27% of plasma levels; therefore concentrations of EPA and DHA in interstitial fluid are expected to be ∼1.7 µM and ∼1.3 µM, respectively, based on the composition of fatty acids in the serum of mice on fatty acid diets [38] and the total amount of fatty acids in serum [39].

Fatty acids have been shown to activate certain GPCRs [40], alter a variety of membrane-mediated cellular functions including platelet aggregation [41] and membrane-bound enzyme activity [42]. These observations along with established effects of fatty acids on BMD provide a strong rationale for the study of the signal transduction pathway of LCPUFA.

To gain insights into the mechanism by which LCPUFA influence bone cells, we have chosen to primarily study activation of the MAPK/ERK pathway via PTH1R, which is known to have an important role in bone remodeling. In this study we test if LCPUFA can: activate the ERKs via PTH1R, modulate PTH hormone signaling, bind to, cause conformational changes and activate PTH1R. Additionally we aimed to determine if (1) a PTH1R antagonist or knockdown of PTH1R is able block PTH1R mediated activation of EPA; (2) if activation of the ERK cascade by LCPUFA is PKA and PKC-dependent; and (3) if LCPUFA play a similar biological survival role as PTH in osteoblasts by examining effects of LCPUFA on Akt phosphorylation and cell survival.

## Results

### Fatty acid induced ERK phosphorylation in MC3T3-E1 and HEK293 cells transfected with PTH1R

**Figure 1:**
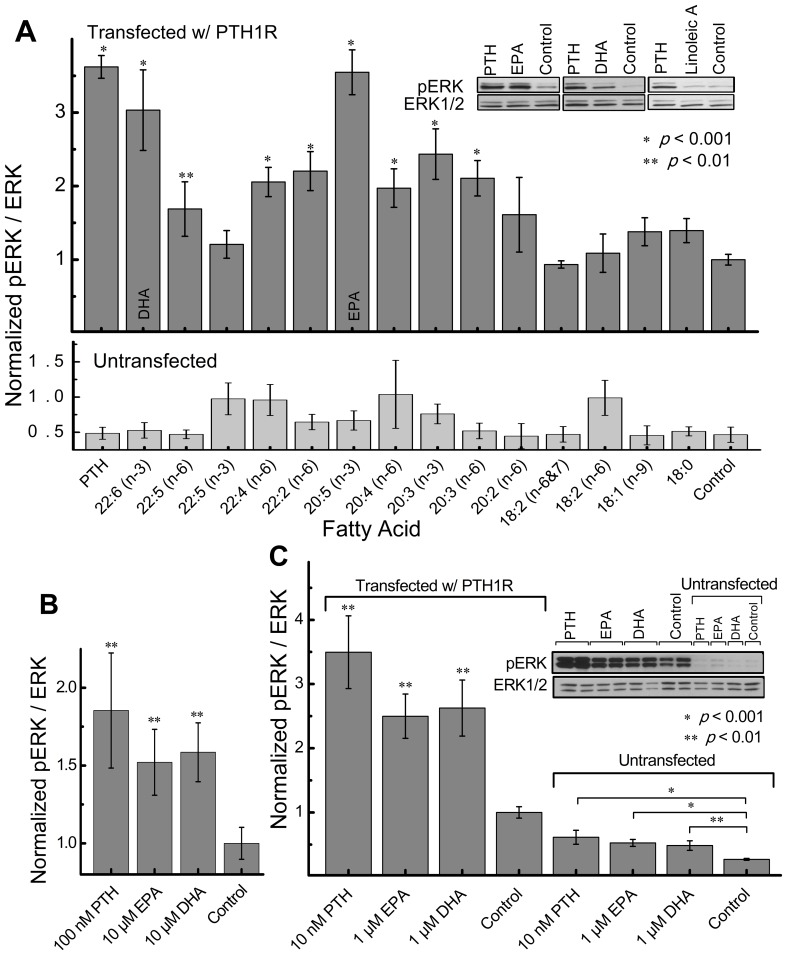
Activation of PTH1R by fatty acids in (A, B) HEK293 and (C) MC3T3-E1 cells. PTH(1–34) concentration in (A, B) was 100 nM (positive control). Fatty acids concentrations were 10 µM. HEK293 cells were transfected with PTH1R as described under Materials & Methods and treated for (A) 5 minutes or (B) 15 seconds. (C) PTH(1–34) concentration was 10 nM (positive control). Fatty acid concentrations were 1 µM. MC3T3-E1 cells were transfected with PTH1R as described under Materials & Methods. Cells were stimulated with PTH(1–34) or corresponding fatty acid in PBS for 5 minutes at 37°C. Western blots were used to determine ERK1/2 phosphorylation. Data represent mean of n≥6 experiments. Error bars indicate standard error of the mean (SEM).

To examine fatty acid activation of the ERK1/2 cascade via PTH1 receptor, we treated PTH1R transfected Human embryonic kidney 293 (HEK293) and Murine calvarial preosteoblast (MC3T3-E1) cells with distinct fatty acids for 5 minutes as shown on Fig. 1. Untransfected cells were also treated with fatty acids as a control (HEK293 do not express endogenous PTH1R [43], [44], whereas differentiated MC3T3-E1 cells do express endogenous PTH1R [45]). In untransfected HEK293 cells, no significant increase in ERK1/2 phosphorylation was found compared to non-treated cells (Fig. 1A; lower panel), indicating that ERK1/2 activation was mediated through the PTH1 receptor. Although untransfected cells were used as controls, transfection with the empty plasmid alone does not alter the ERK phosphorylation levels in HEK293 and MC3T3 cells (Fig. S1). To exclude the possibility that LCPUFA metabolites are activating the ERK pathway, we treated PTH1R transfected HEK293 cells with EPA or DHA for 15 seconds (Fig. 1B). The short treatment also significantly increased ERK1/2 phosphorylation but to a lower magnitude than after the 5 minute treatment; reduced activation was also observed for the PTH treated cells suggesting that the lower ERK activation after the 15 second treatment is due to the intrinsic time scale needed for activation of ERK and not due to the finite production rate of LCPUFA metabolites. Furthermore, the majority of fatty acids have been reported to remain in the plasma membrane after 10 minutes [46] while the LCPUFA metabolizing enzyme, cyclooxygenase is localized in the endoplasmic reticulum [47] making it unlikely that the LCPUFAs metabolites are activating PTH1R in our experiments (in cells, the prostaglandin production rate from EPA is of 4 to 5 hours [48], whereas in vitro the cyclooxygenase reaction kinetics with EPA at nanomolar concentrations is expected to be ∼7 minutes [49]). Differentiated MC3T3-E1 cells also showed a significant response to PTH, EPA, and DHA due to the presence of endogenous PTH1 receptors (Fig. 1C); as expected transfected MC3T3-E1 cells showed even higher response to PTH, EPA and DHA. Note that basal ERK phosphorylation was also increased in the transfected MC3T3-E1 cells due to the higher levels of PTH1 receptor intrinsic constitutive activity and its susceptibility to mechanical perturbation [50] upon handling of the cells. In comparison to the diverse number of fatty acids tested (see Fig. 1A), EPA and DHA induced the highest ERK1/2 phosphorylation with the maximal response (E_max_ = pERK_max_/ERK) of ∼3.5 and ∼3.0 respectively in HEK293 cells.

### PTH1R antagonist blocks EPA induced activation of the ERK cascade

**Figure 2:**
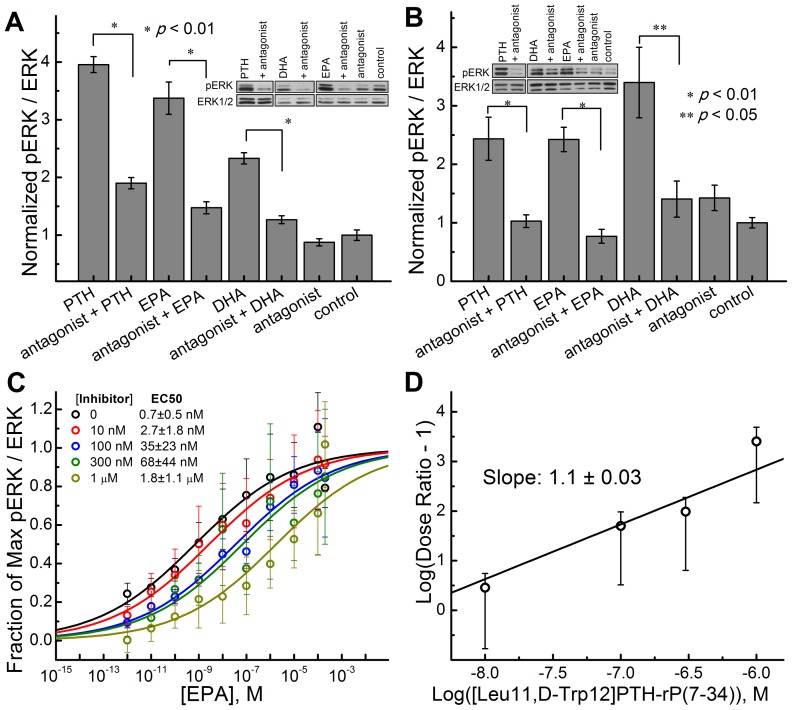
PTH1R antagonist inhibits ERK1/2 response to PTH(1–34) and fatty acids. (A) Effect of 1 µM of PTH1R antagonist [Leu^11^,D-Trp^12^]PTH-rP(7–34) on pERK stimulated with: 10 nM PTH(1–34), 1 µM EPA and 1 µM DHA in DPBS for 5 minutes in HEK293 cells transfected with PTH1R. (B) Effect of 1 µM of PTH1R antagonist, [Nle^8,18^,Tyr^34^]PTH(3–34), on pERK stimulated with 10 nM PTH(1–34), 1 µM EPA, and 1 µM DHA in MC3T3-E1 cells expressing endogenous PTH1R. (C) Dose response to EPA at different concentrations of antagonist [Leu^11^,D-Trp^12^]PTH-rP(7–34) in HEK293 cells. (D) Schild plot of [Leu^11^,D-Trp^12^]PTH-rP(7–34) antagonism on EPA-stimulated PTH1R in HEK293 cells. Experiments were done 24 h after transfection of HEK293 cells with PTH1R in 12-well plates. Ratio of pERK to ERK was measured using western blots. Data represents the mean of at least four independent experiments.

To determine if a PTH1R antagonist can inhibit fatty acid induced ERK phosphorylation, HEK293 cells transfected with PTH1R were treated with EPA or DHA in the presence of PTH1R antagonist, Human [Leu^11^, D-Trp^12^]-PTH-RP (7–34) amide (Fig. 2A). Phospho-ERK immunoblot analysis suggests that the antagonist, Human [Leu^11^, D-Trp^12^]-PTH-RP (7–34) [51], is able to significantly inhibit ERK phosphorylation of cells treated with PTH (1–34) (positive control), EPA and DHA. Similar results were obtained in differentiated MC3T3-E1 cells expressing endogenous PTH1 receptor using another selective PTH1R antagonist, [Nle^8,18^ Tyr^34^]PTH (3–34) which was more efficient at inhibition in MC3T3-E1 cells (Fig. 2B).

### Measurement of antagonist potency of [Leu^11^, D-Trp^12^]-PTH-RP (7–34) on PTH1 receptors expressed in HEK293 cells

To further examine the mechanism of action of antagonist [Leu^11^, D-Trp^12^]-PTH-RP (7–34) on inhibiting PTH1R activation by EPA, we have performed a Schild analysis of the antagonist induced inhibition of ERK activation. [Leu^11^, D-Trp^12^]-PTH-RP (7–34) produced a parallel rightward shift in the EPA concentration dependence curve for stimulation of the ERK cascade via PTH1R activation (Fig. 2C). The antagonist did not significantly affect the maximum possible effect (E_max_) for EPA and did not detectably affect ERK phosphorylation in the absence of the agonist (Fig. 2A,C). The Schild slope was 1.1±0.03 (Fig. 2D). These observations suggest that [Leu^11^, D-Trp^12^]-PTH-RP (7–34) acts as a competitive antagonist of EPA stimulated PTH1R-dependent ERK phosphorylation over the range of antagonist concentrations tested.

### EPA potentiates the effect of PTH

**Figure 3:**
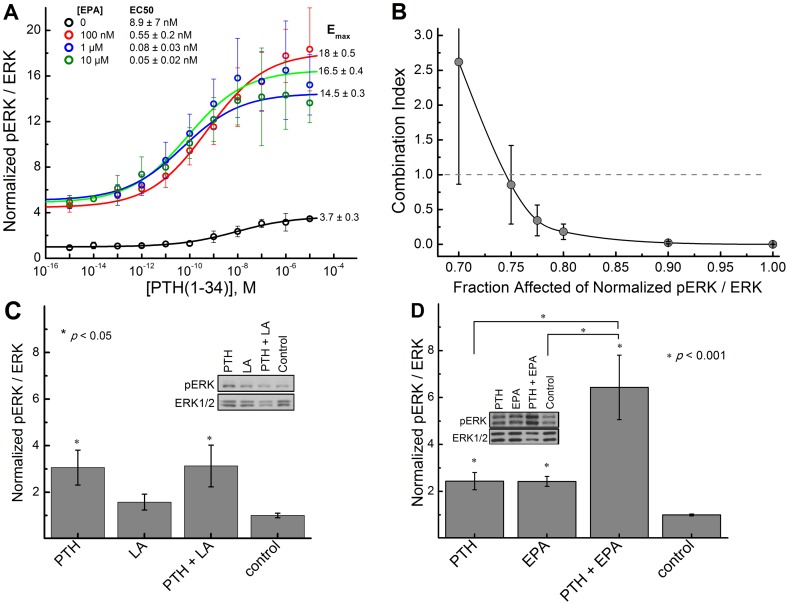
EPA modulates efficacy of PTH(1–34) on ERK1/2 activation. (A) EPA increases the efficacy of PTH(1–34) activation in HEK293 cells. Experiments were done 24 h after transfection of HEK293 cells with PTH1R in 12-well plates. (B) Formal synergy analysis was quantified by combination index analysis versus magnitude of the effect. Combination index values that are less than 1 indicate synergistic interaction. (C) 10 nM PTH(1–34) did not increase the efficacy of 1 µM Linoleic Acid in PTH1R transfected HEK293 cells. (D) 10 nM PTH(1–34) increases the efficacy of 1 µM EPA activation in MC3T3-E1 cells expressing endogenous PTH1R. Ratio of pERK to ERK was measured using western blots. Data represents the mean of at least four independent experiments.

We have further analyzed the effects of EPA on the ability of the cognate ligand, PTH(1–34), to induce ERK1/2 phosphorylation. Figure 3A shows PTH(1–34) dose response curves in the presence of various concentrations of EPA; presented data indicate that (i) the EC50 shifts to lower values at higher EPA concentrations; (ii) the dose response curves are shifted up due to underlying direct EPA-induced stimulation of ERK1/2 at a given EPA concentration; (iii) E_max_ is maximal (at ∼18) at 100 nM and decreases at larger EPA concentrations; and (iv) E_max_ in the presence of EPA is significantly larger than that of PTH(1–34) alone (∼3.5). Our results clearly suggest that EPA potentiates the effect of PTH(1–34) i.e. the cognate ligand PTH(1–34) becomes a superagonist in the presence of EPA (and vice versa). We performed formal synergy analysis using the combination index (CI) approach [52], [53] as described in the Materials and Methods. As the PTH(1–34) and EPA concentrations approach a 1∶1 molar ratio, starting at the fractional effect (*f*_a_) of 0.75 (i.e. activation of ERK cascade at 75%) and higher, the CI value decreases below 1 indicating synergism [52], [53] (Fig. 3B). Moreover, when the *f*_a_ is at 0.9 (which in our experimental data set happens to be when both PTH(1–34) and EPA are at a 1∶1 molar ratio) the CI value is 0.0225 indicating very strong synergism [53]. When the *f*_a_ values are below 0.75 (Fig. 3B), the EPA concentration is 100 fold greater than that of PTH leading to inhibition of PTH binding (see below) and elimination of synergistic effect as a manifested in higher CI value [53]. In contrast, no superagonistic effect is observed in HEK293 cells transfected with PTH1R in response to PTH and 1 µM of the ω-6 Linoleic Acid (Fig. 3C). This superagonistic effect is also seen in MC3T3-E1 cells expressing the endogenous PTH1R receptor (Fig. 3D) since PTH(1–34) is able to significantly increase the response even in the presence of 1 µM of EPA. Data presented in Fig. 3A also show that the relative effect of PTH(1–34) on ERK1/2 phosphorylation (i.e. the relative change in pERK/ERK ratio at saturating PTH(1–34) concentration relative to the baseline value in the absence of PTH(1–34)) does exhibit progressive decrease with increase in EPA concentration (due to displacement of PTH(1–34) by EPA at higher concentrations, see below).

### PKA and PKC inhibitors block EPA induced activation of the ERK cascade

**Figure 4:**
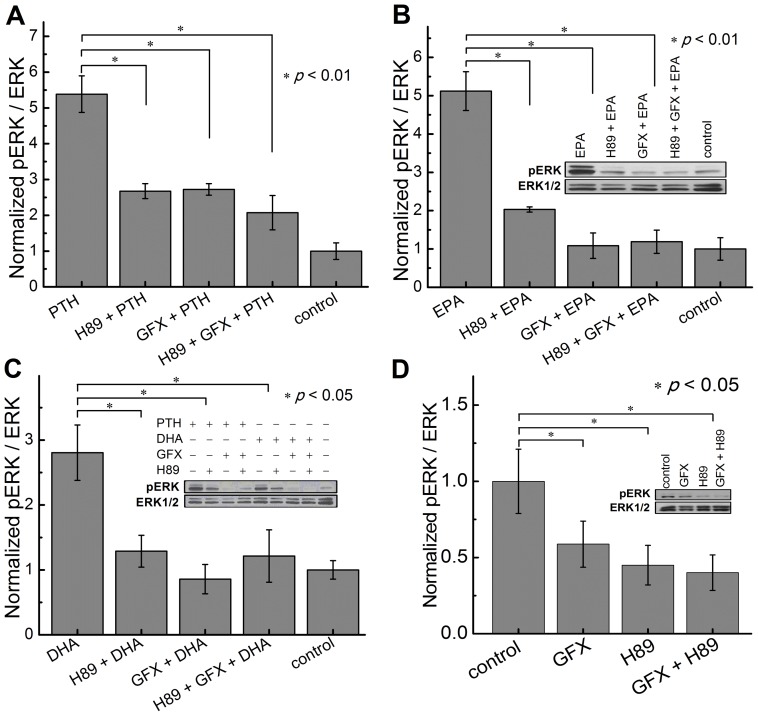
Inhibition of PKA or PKC reduces PTH or fatty acid-stimulated ERK1/2 phosphorylation. (A) HEK293 cells transfected with PTH1R and stimulated with PTH(1–34) (100 nM) for 5 minutes in the absence or presence of H-89 (20 µM), GFX (2.5 µM) or both. Cells were pretreated with inhibitors for 15 minutes. (B) Cells were stimulated with 10 µM EPA. (C) Cells were stimulated with 10 µM DHA. (D) PTH1R transfected cells in the presence of H-89, GFX or both. Values are expressed as ratio of pERK to ERK1/2 after 5 minute stimulation. Data represents the mean from at least three independent experiments.

To gain insight into the ERK activation cascade mechanism, we pre-incubated PTH1R transfected HEK293 cells with either 20 µM of PKA inhibitor, H-89 [54], 2.5 µM of PKC inhibitor, GF109203X [54], or both for 15 minutes prior to agonist treatment; these concentrations have been previously shown to be effective [54] in enabling discrimination between the β-arrestin and G protein–dependent signaling pathway. First we verified that H-89 and GF109203X were able to significantly inhibit PTH(1–34) induced ERK phosphorylation both individually and in combination (Fig. 4A). Both GF109203X and H-89 were able to significantly inhibit EPA and DHA induced ERK phosphorylation (Fig. 4B–C). GF109203X however, was able to inhibit ERK phosphorylation of cells treated with EPA or DHA with a higher potency compared to H-89 at the concentrations used with no additive effect when both inhibitors were used simultaneously (As expected, the PKA and PKC inhibitors were able to decrease the intrinsic activity caused by increased levels of PTH1R (Fig. 4D)).

### Detection of PTH-CC conformational change with EPA

**Figure 5:**
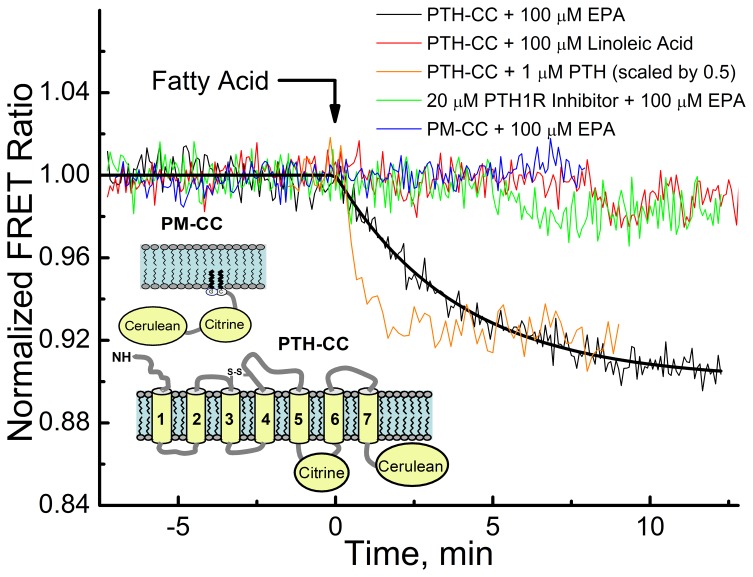
Response of PTH-CC FRET sensor and PM-CC (control FRET sensor) to stimulation by PTH, EPA, EPA and PTH1R inhibitor, [Nle^8,18^,Tyr^34^]PTH(3–34), or linoleic acid. Experiments were done 24 h after transfection of PTH-CC FRET sensor or PM-CC, in HEK293 cells in chambered cover glass at 37°C. FRET ratio was defined as ratio of Citrine emission intensity at 525 nm to Cerulean emission intensity at 475 nm. The FRET response to PTH is from [50].

To test if EPA induces a PTH1R conformational change, we used the previously constructed and characterized PTH1R FRET sensor, PTH-CC, containing an intramolecular FRET pair that enables detection of conformational activity upon ligand stimulation [50]. In the presence of EPA, a decrease in the FRET ratio was observed; whereas EPA treatment of HEK293 cells expressing the control FRET sensor (PM-CC) or treatment of PTH-CC with Linoleic Acid had no effect on the FRET ratio (Fig. 5). Pre-incubation of the PTH-CC transfected HEK293 cells with the selective PTH1R antagonist, [Nle^8,18^, Tyr^34^]PTH(3–34) amide, blocked the conformational response of the PTH1 receptor to EPA. Although GPCR FRET constructs of this type have been shown to have a reduced efficacy of cognate ligands in stimulating conformational changes due to the insertion of the fluorescing proteins into the native receptor structure [50], [55], we were able to observe significant FRET response to LCPUFA.

### Binding characterization of EPA and DHA at PTH1 receptor in HEK293 membrane fractions

To further characterize the association between the fatty acids and the PTH1 receptor, we used the PTH(1–34)^TMR^ fluorescent ligand, where a TMR fluorophore was linked to the lysine side chain (at position 13) of PTH(1–34) as described earlier [56]. Competitive binding experiments using fluorescent anisotropy technique [57] showed that the affinity of PTH(1–34)^TMR^ (*K_i_* = 4.66±4 nM) was close to that of PTH(1–34) for PTH1 receptor (*K_i_* = 2.4±0.1 nM) [56] (Fig. S2; Table 1).

**Table 1:** Summary of binding data.

Receptor	Ligand	IC_50_	*K_i_*
	PTH(1–34)	3.43±0.96 nM	2.4±0.1 nM
PTH1R	PTH(1–34)^TMR^	Not Determined	4.66±4 nM
	EPA	0.96±0.25 nM	0.69±0.25 nM
	DHA	0.76±0.10 nM	0.54±0.16 nM

**Figure 6:**
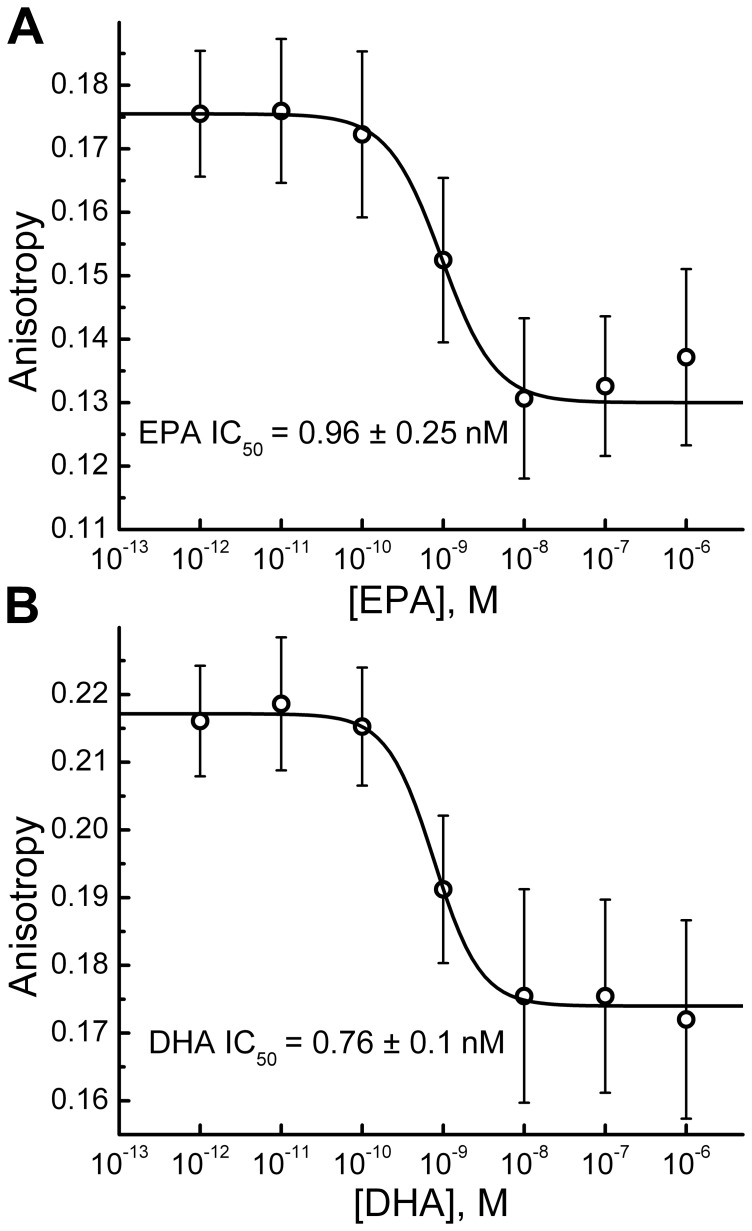
Fluorescence anisotropy of PTH1R membranes labeled with nM PTH(1–34)^TMR^ as function of EPA or DHA concentration. Displacement binding of 2 nM PTH(1–34)^TMR^ with (A) EPA and (B) DHA after 4 hours incubation time. Data represents the mean of at least 15 independent experiments.

To determine the affinity of EPA and DHA to PTH1R, fluorescent ligand, PTH(1–34)^TMR^, at a concentration of 2 nM per well in DPBS was incubated with human PTH1 receptor membrane fractions for four hours at room temperature in the presence of varying concentrations of EPA and DHA. The incubation time we used was based on previous studies in membrane fractions [58] where the dissociation time (t ½ = ∼230 min) of radiolabeled PTH(1–34) was determined . Fluorescent anisotropy was measured using TECAN GENios Pro plate reader. A competitive displacement curve was generated using EPA or DHA as the competitive ligand (Fig. 6A–B). Chen-Prusoff equation [59] was used to convert the obtained IC_50_ to *K_i_* values (Table 1). Both EPA and DHA were able to competitively bind to PTH1 receptor in a dose dependent manner with a *K_i_* of 0.69±0.25 nM and 0.76±0.1 nM respectively.

### PTH1R antagonist blocks EPA-induced activation of the Akt pathway

**Figure 7:**
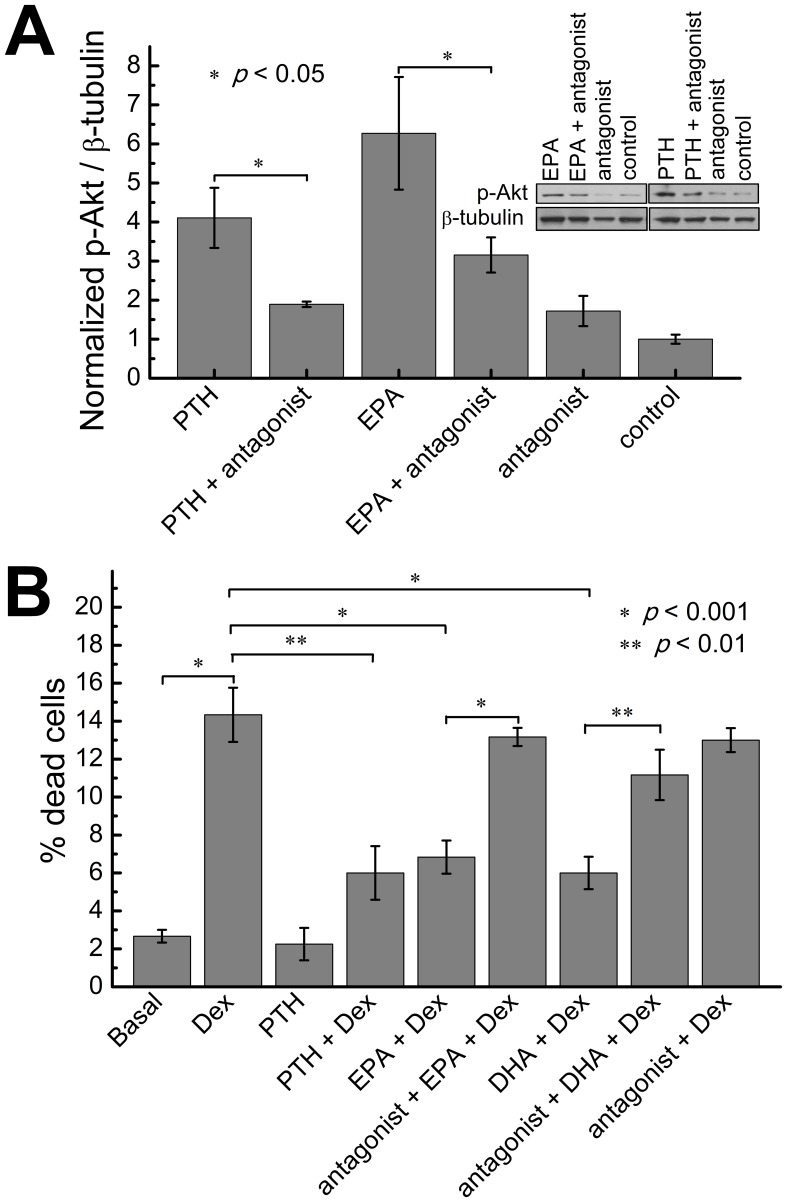
EPA and DHA (A) stimulate Akt phosphorylation and (B) inhibit dexamethasone-induced osteoblast cell death. (A) Phosphorylated Akt levels in MC3T3-E1 cells serum-starved for 4 h and then preincubated with 1 µM of PTH1R antagonist [Leu^11^,D-Trp^12^]PTH-rP(7–34) for 30 minutes followed by 1 µM of LCPUFAs or 10 nM PTH treatment for 5 minutes. (B) Mechanism of the suppressive effect of LCPUFA on cell death in culture of MC3T3-E1 cells. Cultures were maintained for 6 hours in the presence of dexamethasone (Dex) without or with preincubations with 1 µM PTH1R antagonist [Leu^11^,D-Trp^12^]PTH-rP(7–34) for 30 minutes followed by exposure to 1 µM of LCPUFA or 10 nM of PTH for 1 hour. Dead cells were enumerated by trypan blue staining. Data represents the mean of at least 6 independent samples.

To test if LCPUFA similarly to PTH, can modulate biological survival pathway in osteoblasts [11], we characterized changes in Akt phosphorylation in MC3T3 cells treated with EPA. Figure 7A demonstrates an increase in Akt phosphorylation following a 5 minute treatment with EPA. To test if this effect was due to direct PTH1R activation, we pretreated the cells with the selective [Leu^11^, D-Trp^12^]-PTH-RP (7–34) antagonist which lead to a significant reduction EPA-induced Akt phosphorylation.

### PTH1R antagonist blocks EPA-induced osteoblast survival

To investigate if EPA-dependent PTH1R activation has a direct biological effect, we investigated cell survival. Treatment of MC3T3 cells with glucocorticoid, dexamethasone (Dex), induces apoptotic cell death as indicated by an increase in trypan blue staining (Fig. 7B), similarly as reported earlier [60]. Previous reports indicated that the percentage of apoptotic cells determined by trypan blue staining corresponded to that determined by TUNEL staining [61] with at least 90% of trypan blue-stained cells also exhibiting TUNEL labeling [60]. The pro-apoptotic death effect of dexamethasone was attenuated by addition of 10 nM PTH(1–34) as previously reported [60] and by the addition of 1 µM of EPA or DHA. To verify that this effect was specific to PTH1R activation, we pretreated the cells with the selective [Leu^11^, D-Trp^12^]-PTH-RP (7–34) antagonist that significantly reduced the fatty acid survival effect.

### siPTH1R blocks EPA and DHA induced activation of the ERK cascade

**Figure 8:**
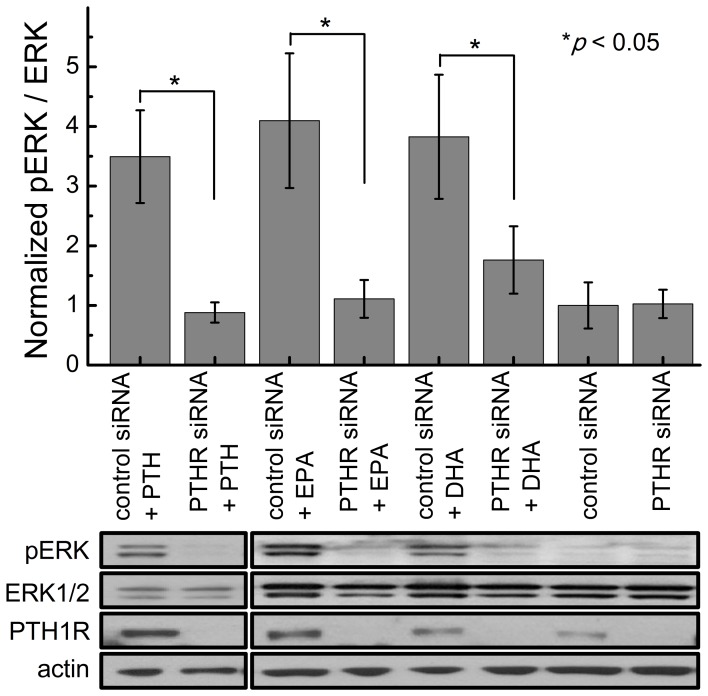
PTH1R siRNA inhibits ERK1/2 response to PTH(1–34) and fatty acids. MC3T3-E1 cells transfected with 100 nM siPTH1R or scambled control siRNA 96 hours prior to treatment with 10 nM PTH(1–34), 1 µM EPA, or 1 µM DHA. Data represents the mean of at least 8 independent samples.

To confirm that LCPUFA induced ERK phosphorylation is unique for and dependent on the PTH1 receptor in differentiated MC3T3 cells, PTH1R was knocked down by siRNA and the effect of LCPUFA were analyzed. MC3T3s were tranfected with 100 nM siPTH1R or scrambled control siRNA for 96 hours prior to treatment. The cells were then treated with either 10 nM PTH(1–34), 1 µM EPA or 1 µM DHA for five minutes. siPTH1R transfected cells had a significant decrease in ERK phosphorylation when treated with PTH, EPA, or DHA compared to the scrambled siRNA transfected cells (Fig. 8).

## Discussion

The results presented in this study indicate that LCPUFA, EPA or DHA, can act as an agonist to the PTH1 receptor at nanomolar concentrations. Our data suggest that the mechanism of activation of PTH1R by EPA involves: binding to the PTH1 receptor, inducing a conformational change similar to that of its cognate ligand (PTH(1–34)), and activation of G protein downstream effectors, PKA and PKC, that ultimately lead to ERK phosphorylation.

Although our primary focus in this study was EPA, which caused the greatest ERK phosphorylation response via PTH1R, DHA and other LCPUFA, both n-3 and n-6, also caused a significant ERK phosphorylation response with somewhat lower potency. To decipher a possible trend in fatty acid induced ERK activation via PTH1R, we tested fatty acids with different carbon lengths and double bond number. Our data presented in Fig. 1 suggest that the length of the fatty acid and the number of carbon chains play a role in PTH1R activation with the exception of docosapentaenoic fatty acid (DPA, 22∶5; n-3). More specifically, our data suggest PTH1R activation requires fatty acids to have an alkyl chain length of no less than 20 carbons with 3 or more double bonds. These results are consistent with other studies in which specific GPCRs have been shown to become activated by length dependent fatty acids [40], [62]–[64]. Several GPCRs have been reported to respond to fatty acids [64], [65]. We have performed dual comparison analysis of PTH1R and the FFA1, FFA2, FFA3, GPR119, and GPR120 fatty acid receptors using the basic local alignment search tool (BLAST). Not one of these receptors had a significant E value (number of matches with same score expected by chance).

The selective PTH1R antagonist or knockdown of PTH1R strongly inhibits the response to EPA or DHA (Fig. 2A,B; 5; 8). To gain further insight into EPA interaction with PTH1R, we were able to produce a rightward-shift of the EPA agonist concentration-dependence curve using a known PTH1R antagonist. This shift had a Schild plot slope of unity and the PTH1R antagonist did not seem to affect the maximal stimulation produced by EPA (Fig. 2C,D). Therefore [Leu^11^, D-Trp^12^]-PTH-RP (7–34) appears to be a pure antagonist to EPA within the detection limits of the assays used.

It has been shown that PTH1R can stimulate ERK1/2 through several distinct signal transduction pathways: a G protein dependent pathway mediated by PKA and PKC and a G protein independent pathway mediated through β-arrestin [54]. The ability of PKA and PKC inhibitors to block EPA and DHA dependent ERK phosphorylation (Fig. 4) suggests that EPA or DHA dependent ERK phosphorylation proceeds predominantly via G_s_ and G_q_ pathways and not through β-arrestin pathway.

Analysis of the presented FRET data (Fig. 5) indicates that EPA treated PTH1R FRET sensor undergoes a similar conformational change (negative change in FRET ratio) as in response to stimulation with its cognate ligand PTH(1–34) [50]. However, the E_max_ of fatty acids on ERK1/2 phosphorylation was somewhat lower as compared to PTH(1–34) (Fig. 1, Fig. 2A) suggesting that the binding of fatty acids stabilize receptor in a state that is different from the one stimulated by the PTH(1–34).

Although in the case of PTH(1–34), intermittent treatment favors bone anabolism and continuous treatment induces the opposite pattern [66], a similar profile has not yet been reported for EPA or other LCPUFAs. EPA can also stimulate other GPCRs that serve other functions. For example, Grp120, a GPCR that binds EPA, mediates potent anti-inflammatory and insulin sensitizing effects [67]. In this study, we primarily focused on PTH1R dependent activation of ERK cascades. ERK is a part of the MAPK signaling component that has been shown to favor osteoblastic cell proliferation and differentiation through transcriptional regulation as well as alkaline phosphatase activity and mineralization [68], [69]. Moreover, PTH1R activation has been shown to lead to an increase in bone formation by prevention of osteoblast apoptosis [60].

Analysis of the presented fluorescence anisotropy data (Fig. 6A–B and Supplementary Information, Fig. S1 online) suggest that EPA and DHA bind to PTH1 receptor in a competitive dose-dependent manner and modulate the affinity of the receptor to its cognate ligand, parathyroid hormone. However, the best hill-fit results in a higher hill coefficient for EPA (1.3±0.6) and DHA (1.5±0.4) as compared to PTH(1–34) (0.63±0.1) indicating that a different binding mechanism is involved in fatty acid binding compared to PTH(1–34). One potential explanation is that the larger hill coefficient suggests a higher degree of cooperativity in fatty acid binding process to PTH1 receptor, further supporting the allosteric nature of the interaction. The fact that rather different fatty acids are able to induce PTH1R signaling (Fig. 1) and competitively displace PTH(1–34) (Fig. 6) suggests that PTH1R receptor may have multiple binding sites that can modulate orthosteric binding site through allosteric interactions or it could respond to changes in lipid bilayer properties induced by LCPUFA. The involvement of allosteric interactions is further supported by superagonistic effect of EPA shown in Fig. 3. Note that the observed decrease of E_max_ at higher EPA concentrations (>100 nM) (Fig. 3) is due to the fact that at higher concentrations, EPA starts to displace PTH(1–34) (as shown in Fig. 6A) leading to reduction in maximal effect caused by PTH(1–34); i.e. maximal superagonism is observed at intermediate EPA concentrations. More detailed investigations will be needed to determine the exact binding sites on the receptor and establish the mechanism by which fatty acids interact with and stimulate PTH1R.

LCPUFAs had similar biological effects as PTH on the Akt and cell survival pathway [11]. It had been shown that glucocorticoid-induced osteoblast apoptosis is mediated by suppression of Akt phosphorylation which can be blocked by PTH [11], [70]. We showed that pretreatment with EPA leads to an increase in PTH1R-dependent Akt phosphorylation. Furthermore, similarly to PTH [11], we showed that LCPUFA were able to significantly inhibit the dexamethasone-induced apoptosis of osteoblastic cells.

The present study reveals the significant effects of fatty acids on PTH1R signaling. In combination, our current findings, numerous previous reports on the importance of PTH signaling on bone homeostasis and the fact that free circulating LCPUFA are present in blood at concentrations used in this study clearly calls for more detailed studies of the role of LCPUFA on PTH pathway both in in-vitro and in-vivo models as it may contribute to development of alternative approaches for treatments of bone diseases such a osteoporosis.

In conclusion, we have identified novel PTH1 receptor agonists, EPA and DHA, which are able to induce PTH1R response with *K_i_* values (0.69±0.25 nM and 0.54±0.25 nM, respectively) similar to cognate agonist PTH(1–34) (∼2.4 nM). Furthermore, we show that other LCPUFAs are also able to activate PTH1 receptor at concentrations of fatty acids typically found in blood. Analysis of the signaling cascade suggest EPA and DHA actions are mediated through PKA and PKC which are known to be downstream signaling molecules of *G_s_* and *G_q_* pathways coupled to PTH1 receptor. The Schild analysis and competitive binding studies suggest that EPA and DHA competitively bind to the PTH1 receptor and EPA acts synergistically with PTH(1–34) causing superagonistic response of ERK1/2. The Akt and cell survival analysis suggests that EPA and DHA may affect osteoblast turnover both by directly activating PTH1R and by modulating the potency of endogenous PTH. In combination, these data provide a molecular basis for the involvement of the PTH1 receptor in mediation of LCPUFAs anabolic effects in bone. Furthermore we believe our study is a starting point for more detailed studies of interactions between fatty acids and other types of GPCRs and more detail understanding of known beneficial effects of fatty acids on human health.

## Materials and Methods

### Cell culture, transfection, and chemicals

HEK293 (American Type Tissue Collection, passages 2–10) cells were grown in DMEM media (Invitrogen, Carlsbad, CA, USA) containing 4.5 g/L D-Glucose and transfected using Targefect-293 (Targeting Systems, Santee, CA, USA). MC3T3-E1 (from American Type Tissue Collection, passages 2–6) cells were cultured in Ascorbic Acid(AA)-free α-modified Eagle's medium (Invitrogen, Carsbad, CA, USA) containing 10% FBS. Differentiation was induced by addition of 50 µg/mL AA. Cells were grown to confluence and treated for 10 days in AA-containing medium. Xfect (Clontech, Mountain View, CA, USA) was used for transfection of MC3T3-E1 cells. Human PTH(1–34) was obtained from Bachem (Torrance, CA, USA). Human [Leu^11^, D-Trp^12^]-PTH-RP (7–34) Amide and [Nle^8,18^ Tyr^34^]PTH (3–34) Amide was purchased from Phoenix Pharmaceuticals, INC (Burlingame, CA, USA). Human [Lys-13(N^ε^-5-carboxy-TMR)]PTH(1–34)NH_2_ [herein termed PTH(1–34)^TMR^] was synthesized by Selleck Chemicals LLC (Houston, TX, USA). Dexamethasone was purchased by Sigma-Aldrich (St. Louis, MO, USA). siRNAs specific for murine PTH1R (1027416) were purchased from Qiagen. Adrenic Acid 22∶4 (n-6) was purchased from Enzo (Plymouth Meeting, PA, USA). Docosahexaenoic Acid 22∶6 (n-3) and Eicosapentaenoic Acid 20∶5 (n-3) were obtained from Cayman Chemical (Ann Arbor, MI, USA). Dihomo-gamma-Linolenic acid 20∶3 (n-6), Docosadienoic Acid 22∶2 (n-6; Nu-Chek-Prep, Elysian, MN), Docosapentaenoic Acid 22∶5 (n-3), Docosapentaenoic Acid 22∶5 (n-6), and Eicosatrienoic Acid 20∶3 (n-3) were purchased from Nu-Chek-Prep (Elysian, MN, USA). Arachidonic Acid 20∶4 (n-6), Conjugated Linoleic Acid 18∶2 (n-6/7), and Eicosadienoic Acid 20∶2 (n-6), were purchased from Matreya (Pleasant Gap, PA, USA) Linoleic Acid 18∶2 (n-6), Oleic Acid 18∶1 (n-9), and Stearic Acid 18∶0 were purchased from Sigma-Aldrich (St. Louis, MO, USA). GF109203X and H-89 were purchased from Enzo (Plymouth Meeting, PA, USA).

### Fatty Acid, PKA and PKC inhibitor Treatment

To examine fatty acid dependent ERK phosphorylation, both MC3T3-E1 and HEK293 cells transfected with PTH1R were treated with various fatty acids containing 0.1% Ethanol and 3.5×10^−5^% BSA in DPBS at 37°C for 5 minutes. The negative control treatment consisted of only 0.1% Ethanol and 3.5×10^−5^% BSA in DPBS at 37°C for 5 minutes. To characterize the effect of PTH1R inhibitor on fatty acid stimulation, HEK293 cells transfected with PTH1R were incubated with either 1 µM, 300 nM, 100 nM, or 10 nM of the PTHR inhibitor, Human [Leu^11^, D-Trp^12^]-PTH-RP (7–34) Amide, for 30 minutes prior to fatty acid treatment; MC3T3-E1 cells were treated with PTH1R inhibitor, Bovine [Nle^8,18^,Tyr^34^]PTH(3–34), prior to fatty acid treatment. To further examine downstream signaling, HEK293 cells transfected with PTH1R were incubated with 20 µM of PKA inhibitor, H-89, or 2.5 µM of PKC inhibitor, GF109203X, for 15 minutes prior to fatty acid treatment.

### Plasma membrane localized FRET sensor (PM-CC)

A control FRET sensor that is localized to the plasma membrane of cells (PM-CC) was constructed as previously described [50]. The construct PM-CC encodes a protein that is comprised of Citrine and Cerulean linked together with a short and flexible GGGGPV (ProVal to encode Age1 restriction site) linker peptide to ensure Förster resonance energy transfer between the two fluorescent proteins, and is fused to another 10 residue leader peptide MGCINSKRKD to direct its translocation to plasma membrane [71].

### PTH1R FRET sensor (PTH-CC)

PTH-CC was constructed and characterized in our previous study as described [50]. In the resultant construct of PTH1R FRET sensor (PTH-CC), the third intracellular loop has the sequence of ATKLRETNAA -Citrine- SCDTRQQYRKLLKST (underlined residues are altered ones to encode restriction sites in the DNA sequences), replacing PTH1R's ATKLRETNAGRCDTRQQYRKLLKST (underlined are the 2 residues where Citrine is inserted in between), and has cytoplasmic COOH terminus fused to Cerulean at residue Ser495 of PTH1R with insertion of two extra residues encoding Pro-Val to accommodate an *Age*1 restriction site. The sequences of all constructs were confirmed by sequencing service.

### FRET measurements

FRET measurements in single living cells, 24 hours after being transfected, were performed using multichannel time resolved single photon counting as previously described [50], [72]. Briefly, fluorescence emission kinetics and spectra were measured by using a multichannel, time-correlated single photon counting spectrograph (PML-16/SPC630; Becker & Hickl, Berlin, Germany) coupled to an inverted microscope (Axiovert 200 M; Zeiss, Thornwood, NY) via fiber optic link. A femtosecond Ti:Sapphire oscillator (Spectra-Physics, Irvine, CA, USA) was used as the excitation source. The repetition frequency of the light pulses from the oscillator was reduced to 8 MHz and the wavelength was doubled to 435 nm. The excitation light was defocused to a spot size of 20–50 µm to enable spatially homogenous excitation of a single cell. Single cell fluorescence spectra were obtained by integrating time-resolved fluorescence data. Presented data were recorded by detecting fluorescence emission polarized at the magic angle (54.7°) to the polarization of the excitation light at 435 nm to reduce contributions from potential reorientation of the sensor in the plasma membrane under shear stress. FRET ratio was defined as ratio of Citrine emission intensity at ∼525 nm to Cerulean emission intensity at 475 nm. Duration FRET measurements were kept to a minimum to prevent photobleaching.

### Western blot analysis

Both MC3T3-E1 and HEK293 cells were washed with ice cold DPBS, collected, and lysed in lysis buffer containing 50 mM Tris –HCl, 135 mM NaCl, 60 mM n-octyl beta-D-glucopyranoside (EMD Chemicals, Gibbstown, NJ, USA), protease inhibitor cocktail, and phospahtase inhibitor cocktail (Roche, Basel, Switzerland) for 30 minutes on ice. SDS sample buffer was added to the lysate and incubated at 95°C for 5 min. Cell extracts were resolved by SDS-PAGE and transferred to polyvinylidine difluoride (PVDF) membranes. Blots were probed with anti-phospho-p44/42 MAPK (Erk1/2 rabbit polyclonal), anti-p44/42 MAPK (Erk1/2 rabbit polyclonal), and anti-phopho-Akt (S473; Cell Signaling Technology, Beverly, MA, USA). Beta-tubulin (H-235) was from Santa Cruz Biotechnology. Immunoreactive bands were detected with the appropriate horseradish peroxidase-conjugated secondary antibodies (anti-rabbit; Cell Signaling Technology, Beverly, MA, USA, 7074) and visualized by enhanced chemiluminescence (Thermo Scientific, Rockford, IL, USA). The band intensities were measured by densitometry analysis using Image J (NIH, Bethesda, MD, USA) and the change in phosphorylated ERK in both cell lines was calculated as the pERK/ERK ratio. The change in phosphorylated Akt was calculated as the pAkt/beta-tubulin ratio.

### Preparation of cell membranes

Membrane fractions were prepared using confluent monolayers of HEK293 cells transfected with PTH1R in 10-cm plates. Cells were lifted and centrifuged at 1000 *g* for 5 minutes. Cell pellet was resuspended in 1 mL DPBS with protease inhibitor cocktail (Roche, Basel, Switzerland) and 0.5 mm Zirconia/Silica Beads (BioSpec Products, Bartlesville, OK, USA). The sample was homogenized by vortexing at 4°C for 10 minutes. The homogenate was then centrifuged at 1000 *g* for 5 minutes to remove unbroken cells and larger debris. The supernatant was centrifuged at 50,000 *g* at 4°C for 60 minutes (Beckman L-80 ultracentrifuge, SW-56 rotor) and the pellet resuspended in DPBS. Cell membrane fractions were stored at −80°C.

### Fluorescence Anisotropy

Fluorescence anisotropy was measured on TECAN GENios Pro (Tecan Group Ltd. Männedorf, Switzerland) according to the equation, 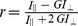, where 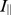 is the parallel emission and 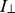 is the perpendicular emission with respect to the excitation and *G* is the calibration factor for the plate reader. Spectral filters, polarizers, and dichroic mirrors were either factory supplied or obtained from CVI Melles Griot (Carlsbad, CA, USA). Excitation filter 527 nm±5 nm, emission filter 600 nm±20 nm, and dichroic-2 mirror. The measurements were recorded using 25 excitation flashes with a 40 µs integration time in black flat bottom 96-well plates (Greiner Bio-One, Monroe, NC, USA). In order to have a consistent surface tension and reduce the effect of increased peptide concentration on the meniscus in each well, the plates were blocked with 10% non-fat milk for 2 hours and washed with DPBS prior to start of assay. Total assay volume was 150 µL, obtained by the sequential addition of 80 µL of buffer (DPBS), 5 µL of membrane preparation, 15 µL of competitive displacing ligand, and 50 µL of fluorescent tracer PTH(1–34)^TMR^ .

### Determination of osteoblast cell death

Cell death was quantified after combining nonadherent and adherent cells released from the culture dish using trypsin-EDTA. Cells were resuspended in phosphate-buffered saline (PBS) containing 0.5% bovine serum albumin (BSA) prior to analysis. For determination of dead cells by dye exclusion, 0.4% Trypan Blue (Sigma-Aldrich St. Louis, MO, USA) was added, and the percentage of cells exhibiting both nuclear and cytoplasmic trypan blue staining was determined using a hemocytometer. A minimum of 100 cells were counted.

### Statistical analyses

Statistical significance was evaluated for differences between groups from at least three independent experiments using Student's *t*-test. A *p* value of <0.05 was considered to be statistically significant. Calculations of the mean, standard error of the mean,and Student's *t*-test were performed using Microsoft Excel and OriginPro (OriginLab Co., Northampton, MA).

### Combination Index analysis

Synergy was characterized using combination index [53] calculated as: 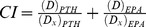, where CI<1,  = 1, and >1 indicate synergism, additive effect, and antagonism, respectively. D is a concentration of either PTH(1–34) or EPA. In the denominator, (D_x_) is for D_PTH_ alone and (D_x_)_EPA_ is for D_EPA_ alone that can activate the ERK pathway to the factional effect *x*. In the numerator, (D)_PTH_+(D)_PTH_ is a given combination of PTH and EPA that activates the ERK pathway to the same fractional effect *x*. Concentration values were obtained using data from Figure 2C and Figure 3A and utilizing synergy analysis methods of Chou and Talalay [52].

## Supporting Information

Figure S1 **pERK levels in HEK393 and MC3T3 untransfected or transfected with the empty plasmid pcDNA3.1.** Data represents mean ± SEM of at least 3 independent experiments.
(DOCX)

Figure S2 **Fluorescence anisotropy of PTH1R membranes labeled with nM PTH(1–34)^TMR^ as function of PTH(1–34) concentration after 4 hours incubation at room temperature.** Data represents mean ± SEM of at least 15 independent experiments.
(DOCX)

## References

[1] Ensrud KE, Palermo L, Black DM, Cauley J, Jergas M, et al (1995) Hip and calcaneal bone loss increase with advancing age: Longitudinal results from the study of osteoporotic fractures. J Bone Miner Res 10: 1778–1787. 8592956 10.1002/jbmr.5650101122

[2] Rossignol M, Moride Y, Perreault S, Boivin JF, Ste-Marie LG, et al (2002) Recommendations for the prevention of osteoporosis and fragility fractures. International comparison and synthesis. Int J Technol Assess Health Care 18: 597–610. 12391952

[3] Abellan van Kan G, Gambassi G, de Groot LC, Andrieu S, Cederholm T, et al (2008) Nutrition and aging. The Carla Workshop. The journal of nutrition, health & aging 12: 355–364. 10.1007/BF02982667 PMC12892701 18548172

[4] Poole KE, Reeve J (2005) Parathyroid hormone - a bone anabolic and catabolic agent. Curr Opin Pharmacol 5: 612–617. 16181808 10.1016/j.coph.2005.07.004

[5] Juppner H, Gardella T, Kronenberg H, Potts J Jr (2001) Endocrinology (DeGroot L, and Jameson J, eds). 2: : 969–1053.

[6] Strewler GJ (2000) The physiology of parathyroid hormone-related protein. N Engl J Med 342: 177–185. 10639544 10.1056/NEJM200001203420306

[7] Mahon MJ, Bonacci TM, Divieti P, Smrcka AV (2006) A docking site for G protein betagamma subunits on the parathyroid hormone 1 receptor supports signaling through multiple pathways. Mol endocrinol 20: 136–146. 16099817 10.1210/me.2005-0169

[8] Bastepe M, Weinstein LS, Ogata N, Kawaguchi H, Juppner H, et al (2004) Stimulatory G protein directly regulates hypertrophic differentiation of growth plate cartilage in vivo. Proc Natl Acad Sci U S A 101: 14794–14799. 15459318 10.1073/pnas.0405091101 PMC522030

[9] Mahon MJ, Donowitz M, Yun CC, Segre GV (2002) Na(+)/H(+) exchanger regulatory factor 2 directs parathyroid hormone 1 receptor signalling. Nature 417: 858–861. 12075354 10.1038/nature00816

[10] Datta NS, Abou-Samra AB (2009) PTH and PTHrP signaling in osteoblasts. Cell Signal 21: 1245–1254. 19249350 10.1016/j.cellsig.2009.02.012 PMC2723940

[11] Weinstein RS, Jilka RL, Almeida M, Roberson PK, Manolagas SC (2010) Intermittent parathyroid hormone administration counteracts the adverse effects of glucocorticoids on osteoblast and osteocyte viability, bone formation, and strength in mice. Endocrinology 151: 2641–2649. 20410195 10.1210/en.2009-1488 PMC2875832

[12] Mukherjee A, Rotwein P (2009) Akt promotes BMP2-mediated osteoblast differentiation and bone development. J Cell Sci 122: 716–726. 19208758 10.1242/jcs.042770 PMC2720922

[13] Datta SR, Brunet A, Greenberg ME (1999) Cellular survival: a play in three Akts. Genes Dev 13: 2905–2927. 10579998 10.1101/gad.13.22.2905

[14] Watkins BA, Lippman HE, Le Bouteiller L, Li Y, Seifert MF (2001) Bioactive fatty acids: role in bone biology and bone cell function. Prog Lipid Res 40: 125–148. 11137570 10.1016/s0163-7827(00)00016-3

[15] Fernandes G, Bhattacharya A, Rahman M, Zaman K, Banu J (2008) Effects of n-3 fatty acids on autoimmunity and osteoporosis. Front Biosci 13: 4015–4020. 18508495 10.2741/2989

[16] Lavie CJ, Milani RV, Mehra MR, Ventura HO (2009) Omega-3 polyunsaturated fatty acids and cardiovascular diseases. J Am Coll Cardiol 54: 585–594. 19660687 10.1016/j.jacc.2009.02.084

[17] Poulsen RC, Moughan PJ, Kruger MC (2007) Long-chain polyunsaturated fatty acids and the regulation of bone metabolism. Exp Biol Med (Maywood) 232: 1275–1288. 17959840 10.3181/0704-MR-100

[18] Coetzee M, Haag M, Joubert AM, Kruger MC (2007) Effects of arachidonic acid, docosahexaenoic acid and prostaglandin E(2) on cell proliferation and morphology of MG-63 and MC3T3-E1 osteoblast-like cells. Prostaglandins Leukot Essent Fatty Acids 76: 35–45. 17113274 10.1016/j.plefa.2006.10.001

[19] Bonnet N, Ferrari SL (2011) Effects of long-term supplementation with omega-3 fatty acids on longitudinal changes in bone mass and microstructure in mice. J Nutr Biochem 22: 665–672. 21036590 10.1016/j.jnutbio.2010.05.006

[20] Sakaguchi K, Morita I, Murota S (1994) Eicosapentaenoic acid inhibits bone loss due to ovariectomy in rats. Prostaglandins Leukot Essent Fatty Acids 50: 81–84. 8171071 10.1016/0952-3278(94)90151-1

[21] Kruger MC, Coetzer H, de Winter R, Gericke G, van Papendorp DH (1998) Calcium, gamma-linolenic acid and eicosapentaenoic acid supplementation in senile osteoporosis. Aging 10: 385–394. 9932142 10.1007/BF03339885

[22] Shichikawa K, Takenaka Y, Maeda A, Yoshino R, Tsujimoto M, et al (1981) A longitudinal population survey of rheumatoid arthritis in a rural district in Wakayama. Ryumachi [Rheumatism] 21 Suppl: 35–43. 7344133

[23] Zwart SR, Pierson D, Mehta S, Gonda S, Smith SM (2010) Capacity of Omega-3 Fatty Acids or Eicosapentaenoic Acid to Counteract Weightlessness-Induced Bone Loss by Inhibiting NF-kappa B Activation: From Cells to Bed Rest to Astronauts. J Bone Miner Res 25: 1049–1057. 19874203 10.1359/jbmr.091041

[24] Hogstrom M, Nordstrom P, Nordstrom A (2007) n-3 fatty acids are positively associated with peak bone mineral density and bone accrual in healthy men: the NO2 Study. Am J Clin Nutr 85: 803–807. 17344503 10.1093/ajcn/85.3.803

[25] Weiss LA, Barrett-Connor E, von Muhlen D (2005) Ratio of n-6 to n-3 fatty acids and bone mineral density in older adults: the Rancho Bernardo study. Am J Clin Nutr 81: 934–938. 15817874 10.1093/ajcn/81.4.934

[26] Kruger MC, Coetzer H, de Winter R, Claassen N (1995) Eicosapentaenoic acid and docosahexaenoic acid supplementation increases calcium balance. Nutr Res 15: 211–219.

[27] Schlemmer CK, Coetzer H, Claassen N, Kruger MC (1999) Oestrogen and essential fatty acid supplementation corrects bone loss due to ovariectomy in the female Sprague Dawley rat. Prostaglandins Leukot Essent Fatty Acids 61: 381–390. 10718112 10.1054/plef.1999.0116

[28] Li Y, Seifert MF, Lim SY, Salem N, Watkins BA (2010) Bone mineral content is positively correlated to n-3 fatty acids in the femur of growing rats. Brit J Nutr 104: 674–685. 20420751 10.1017/S0007114510001133

[29] Hamilton JA, Kamp F (1999) How are free fatty acids transported in membranes? Is it by proteins or by free diffusion through the lipids? Diabetes 48: 2255–2269. 10580412 10.2337/diabetes.48.12.2255

[30] Salem N Jr, Kim HY, and Yergey JA (1986) Docosahexaenoic Acid: Membrane Function and Metabolism. In: Simopoulos AP, Kifer RR, and Martin RE, editors. Health Effects of Polyunsaturated Fatty Acids in Seafoods. Academic Press: Academic Press. pp. 263–317.

[31] VanMeter AR, Ehringer WD, Stillwell W, Blumenthal EJ, Jenski LJ (1994) Aged lymphocyte proliferation following incorporation and retention of dietary omega-3 fatty acids. Mech Ageing Dev 75: 95–114. 7529860 10.1016/0047-6374(94)90079-5

[32] Robinson DR, Xu LL, Knoell CT, Tateno S, Olesiak W (1993) Modification of spleen phospholipid fatty acid composition by dietary fish oil and by n-3 fatty acid ethyl esters. J Lipid Res 34: 1423–1434. 8409773

[33] Albert CM, Campos H, Stampfer MJ, Ridker PM, Manson JE, et al (2002) Blood levels of long-chain n-3 fatty acids and the risk of sudden death. N Engl J Med 346: 1113–1118. 11948270 10.1056/NEJMoa012918

[34] Leone TC, Weinheimer CJ, Kelly DP (1999) A critical role for the peroxisome proliferator-activated receptor alpha (PPARalpha) in the cellular fasting response: the PPARalpha-null mouse as a model of fatty acid oxidation disorders. Proc Natl Acad Sci U S A 96: 7473–7478. 10377439 10.1073/pnas.96.13.7473 PMC22110

[35] Fan YY, McMurray DN, Ly LH, Chapkin RS (2003) Dietary (n-3) polyunsaturated fatty acids remodel mouse T-cell lipid rafts. J Nutr 133: 1913–1920. 12771339 10.1093/jn/133.6.1913

[36] Owen M, Triffitt JT (1976) Extravascular albumin in bone tissue. J Physiol 257: 293–307. 950595 10.1113/jphysiol.1976.sp011369 PMC1309360

[37] McCarthy ID (1997) Clearance of albumin by cortical bone and marrow. Clin Orthop Relat Res 24–29. 9005892

[38] Sun D, Krishnan A, Zaman K, Lawrence R, Bhattacharya A, et al (2003) Dietary n-3 fatty acids decrease osteoclastogenesis and loss of bone mass in ovariectomized mice. J Bone Miner Res 18: 1206–1216. 12854830 10.1359/jbmr.2003.18.7.1206

[39] Moitra J, Mason MM, Olive M, Krylov D, Gavrilova O, et al (1998) Life without white fat: a transgenic mouse. Genes Dev 12: 3168–3181. 9784492 10.1101/gad.12.20.3168 PMC317213

[40] Wang J, Wu X, Simonavicius N, Tian H, Ling L (2006) Medium-chain fatty acids as ligands for orphan G protein-coupled receptor GPR84. J Biol Chem 281: 34457–34464. 16966319 10.1074/jbc.M608019200

[41] Hoak JC, Spector AA, Fry GL, Warner ED (1970) Effect of free fatty acids on ADP-induced platelet aggregation. Nature 228: 1330–1332. 5488112 10.1038/2281330a0

[42] Anderson WB, Jaworski CJ (1977) Modulation of adenylate cyclase activity of fibroblasts by free fatty acids and phospholipids. Arch Biochem Biophys 180: 374–383. 560173 10.1016/0003-9861(77)90051-0

[43] Thomas P, Smart TG (2005) HEK293 cell line: a vehicle for the expression of recombinant proteins. J Pharmacol Toxicol Methods 51: 187–200. 15862464 10.1016/j.vascn.2004.08.014

[44] Sneddon WB, Yang YM, Ba JM, Harinstein LM, Friedman PA (2007) Extracellular signal-regulated kinase activation by parathyroid hormone in distal tubule cells. Am J Physiol-Renal 292: F1028–F1034. 10.1152/ajprenal.00288.2006 17107942

[45] Wang D, Christensen K, Chawla K, Xiao G, Krebsbach PH, et al (1999) Isolation and characterization of MC3T3-E1 preosteoblast subclones with distinct in vitro and in vivo differentiation/mineralization potential. J Bone Miner Res 14: 893–903. 10352097 10.1359/jbmr.1999.14.6.893

[46] Klausner RD, Kleinfeld AM, Hoover RL, Karnovsky MJ (1980) Lipid domains in membranes. Evidence derived from structural perturbations induced by free fatty acids and lifetime heterogeneity analysis. J Biol Chem 255: 1286–1295. 7354027

[47] Otto JC, Smith WL (1994) The orientation of prostaglandin endoperoxide synthases-1 and -2 in the endoplasmic reticulum. J Biol Chem 269: 19868–19875. 8051068

[48] Saito J, Terano T, Hirai A, Shiina T, Tamura Y, et al (1997) Mechanisms of enhanced production of PGI2 in cultured rat vascular smooth muscle cells enriched with eicosapentaenoic acid. Atherosclerosis 131: 219–228. 9199275 10.1016/s0021-9150(97)00048-8

[49] Kulmacz RJ, Pendleton RB, Lands WE (1994) Interaction between peroxidase and cyclooxygenase activities in prostaglandin-endoperoxide synthase. Interpretation of reaction kinetics. J Biol Chem 269: 5527–5536. 8119886

[50] Zhang YL, Frangos JA, Chachisvilis M (2009) Mechanical stimulus alters conformation of type 1 parathyroid hormone receptor in bone cells. Am J Physiol-Cell Ph 296: C1391–C1399. 10.1152/ajpcell.00549.2008 PMC2692421 19369447

[51] Nutt RF, Caulfield MP, Levy JJ, Gibbons SW, Rosenblatt M, et al (1990) Removal of partial agonism from parathyroid hormone (PTH)-related protein-(7–34)NH2 by substitution of PTH amino acids at positions 10 and 11. Endocrinology 127: 491–493. 2163325 10.1210/endo-127-1-491

[52] Chou TC, Talalay P (1984) Quantitative analysis of dose-effect relationships: the combined effects of multiple drugs or enzyme inhibitors. Adv Enzyme Regul 22: 27–55. 6382953 10.1016/0065-2571(84)90007-4

[53] Chou TC (2006) Theoretical basis, experimental design, and computerized simulation of synergism and antagonism in drug combination studies. Pharmacol Rev 58: 621–681. 16968952 10.1124/pr.58.3.10

[54] Gesty-Palmer D, Chen M, Reiter E, Ahn S, Nelson CD, et al (2006) Distinct beta-arrestin- and G protein-dependent pathways for parathyroid hormone receptor-stimulated ERK1/2 activation. J Biol Chem 281: 10856–10864. 16492667 10.1074/jbc.M513380200

[55] Vilardaga JP, Steinmeyer R, Harms GS, Lohse MJ (2005) Molecular basis of inverse agonism in a G protein-coupled receptor. Nat Chem Biol 1: 25–28. 16407989 10.1038/nchembio705

[56] Castro M, Nikolaev VO, Palm D, Lohse MJ, Vilardaga JP (2005) Turn-on switch in parathyroid hormone receptor by a two-step parathyroid hormone binding mechanism. Proc Natl Acad Sci U S A 102: 16084–16089. 16236727 10.1073/pnas.0503942102 PMC1276049

[57] Rossi AM, Taylor CW (2011) Analysis of protein-ligand interactions by fluorescence polarization. Nat Protoc 6: 365–387. 21372817 10.1038/nprot.2011.305 PMC3160472

[58] Dean T, Linglart A, Mahon MJ, Bastepe M, Juppner H, et al (2006) Mechanisms of ligand binding to the parathyroid hormone (PTH)/PTH-related protein receptor: selectivity of a modified PTH(1–15) radioligand for GalphaS-coupled receptor conformations. Mol Endocrinol 20: 931–943. 16339275 10.1210/me.2005-0349 PMC3242416

[59] Cheng HC (2001) The power issue: determination of KB or Ki from IC50. A closer look at the Cheng-Prusoff equation, the Schild plot and related power equations. J Pharmacol Toxicol Methods 46: 61–71. 12481843 10.1016/s1056-8719(02)00166-1

[60] Jilka RL, Weinstein RS, Bellido T, Roberson P, Parfitt AM, et al (1999) Increased bone formation by prevention of osteoblast apoptosis with parathyroid hormone. J Clin Invest 104: 439–446. 10449436 10.1172/JCI6610 PMC408524

[61] Jilka RL, Weinstein RS, Bellido T, Parfitt AM, Manolagas SC (1998) Osteoblast programmed cell death (apoptosis): modulation by growth factors and cytokines. J Bone Miner Res 13: 793–802. 9610743 10.1359/jbmr.1998.13.5.793

[62] Brown AJ, Goldsworthy SM, Barnes AA, Eilert MM, Tcheang L, et al (2003) The Orphan G protein-coupled receptors GPR41 and GPR43 are activated by propionate and other short chain carboxylic acids. J Biol Chem 278: 11312–11319. 12496283 10.1074/jbc.M211609200

[63] Le Poul E, Loison C, Struyf S, Springael JY, Lannoy V, et al (2003) Functional characterization of human receptors for short chain fatty acids and their role in polymorphonuclear cell activation. J Biol Chem 278: 25481–25489. 12711604 10.1074/jbc.M301403200

[64] Hirasawa A, Tsumaya K, Awaji T, Katsuma S, Adachi T, et al (2005) Free fatty acids regulate gut incretin glucagon-like peptide-1 secretion through GPR120. Nat Med 11: 90–94. 15619630 10.1038/nm1168

[65] Stoddart LA, Smith NJ, Milligan G (2008) International Union of Pharmacology. LXXI. Free fatty acid receptors FFA1, -2, and -3: pharmacology and pathophysiological functions. Pharmacol Rev 60: 405–417. 19047536 10.1124/pr.108.00802

[66] Qin L, Raggatt LJ, Partridge NC (2004) Parathyroid hormone: a double-edged sword for bone metabolism. Trends Endocrinol Metab 15: 60–65. 15036251 10.1016/j.tem.2004.01.006

[67] Oh DY, Talukdar S, Bae EJ, Imamura T, Morinaga H, et al (2010) GPR120 is an omega-3 fatty acid receptor mediating potent anti-inflammatory and insulin-sensitizing effects. Cell 142: 687–698. 20813258 10.1016/j.cell.2010.07.041 PMC2956412

[68] Xiao G, Jiang D, Thomas P, Benson MD, Guan K, et al (2000) MAPK pathways activate and phosphorylate the osteoblast-specific transcription factor, Cbfa1. J Biol Chem 275: 4453–4459. 10660618 10.1074/jbc.275.6.4453

[69] Sowa H, Kaji H, Yamaguchi T, Sugimoto T, Chihara K (2002) Activations of ERK1/2 and JNK by transforming growth factor beta negatively regulate Smad3-induced alkaline phosphatase activity and mineralization in mouse osteoblastic cells. J Biol Chem 277: 36024–36031. 12130649 10.1074/jbc.M206030200

[70] Smith E, Frenkel B (2005) Glucocorticoids inhibit the transcriptional activity of LEF/TCF in differentiating osteoblasts in a glycogen synthase kinase-3beta-dependent and -independent manner. J Biol Chem 280: 2388–2394. 15537647 10.1074/jbc.M406294200

[71] Zacharias DA, Violin JD, Newton AC, Tsien RY (2002) Partitioning of lipid-modified monomeric GFPs into membrane microdomains of live cells. Science 296: 913–916. 11988576 10.1126/science.1068539

[72] Chachisvilis M, Zhang YL, Frangos JA (2006) G protein-coupled receptors sense fluid shear stress in endothelial cells. Proc Natl Acad Sci U S A 103: 15463–15468. 17030791 10.1073/pnas.0607224103 PMC1622845

