# Supplementary material for: PTH1 Receptor Is Involved in Mediating Cellular Response to Long-Chain Polyunsaturated Fatty Acids
Source: PLoS One. 2012 Dec 27;7(12):e52583. doi: 10.1371/journal.pone.0052583 (PMC3531455; doi:10.1371/journal.pone.0052583)
Supplement: Figure S1 — pERK levels in HEK393 and MC3T3 untransfected or transfected with the empty plasmid pcDNA3.1. Data represents mean ± SEM of at least 3 independent experiments. (DOCX) [file pone.0052583.s001.docx]

**
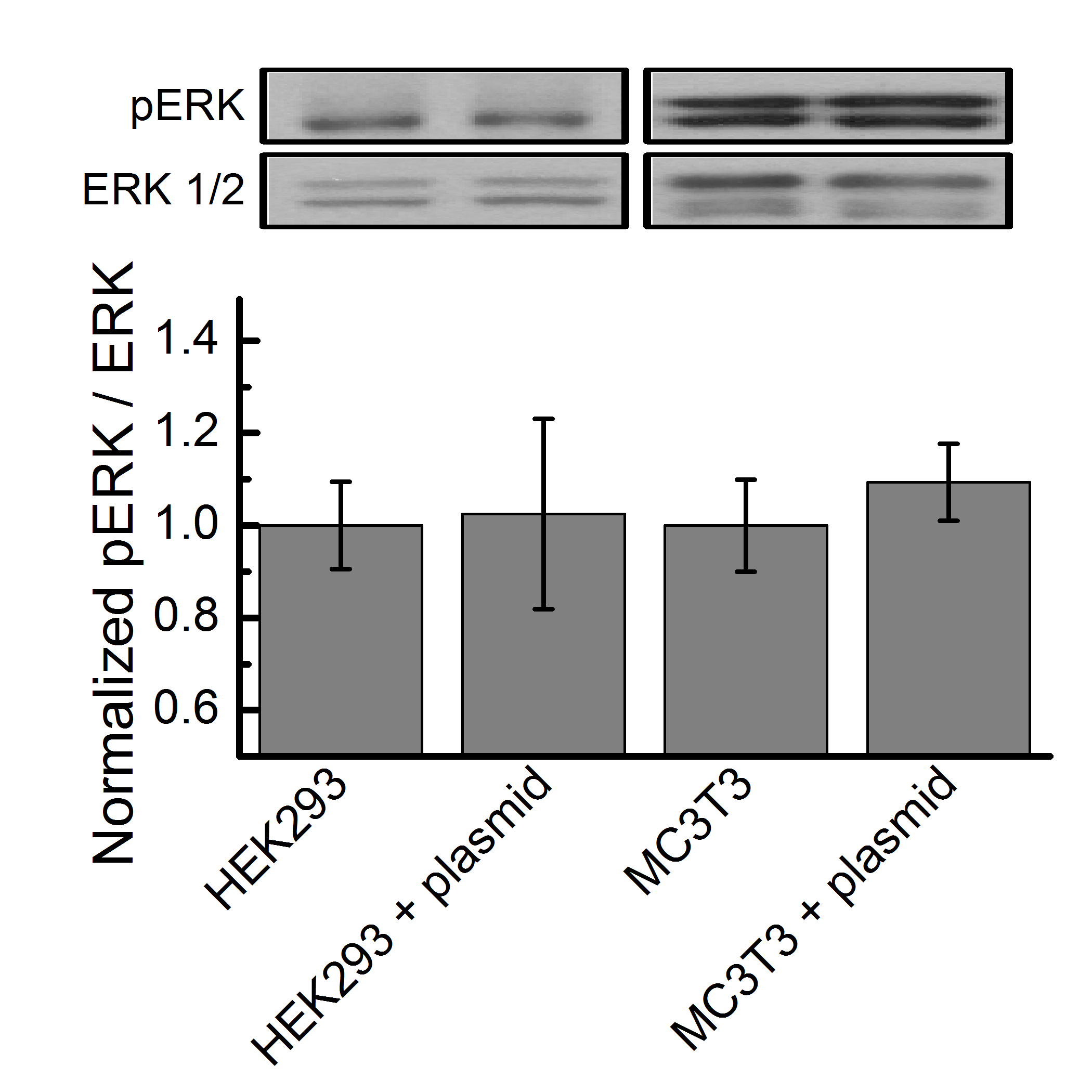
**

**Figure S1.** pERK levels in HEK393 and MC3T3 untransfected or transfected with the empty plasmid pcDNA3.1. Data represents mean ± SEM of at least 3 independent experiments.
