# Supplementary material for: PTH1 Receptor Is Involved in Mediating Cellular Response to Long-Chain Polyunsaturated Fatty Acids
Source: PLoS One. 2012 Dec 27;7(12):e52583. doi: 10.1371/journal.pone.0052583 (PMC3531455; doi:10.1371/journal.pone.0052583)
Supplement: Figure S2 — Fluorescence anisotropy of PTH1R membranes labeled with nM PTH(1–34)TMR as function of PTH(1–34) concentration after 4 hours incubation at room temperature. Data represents mean ± SEM of at least 15 independent experiments. (DOCX) [file pone.0052583.s002.docx]

**

**

**Figure S2.** Fluorescence anisotropy of PTH1R membranes labeled with nM PTH(1-34)^TMR^ as function of PTH(1-34) concentration after 4 hours incubation at room temperature. Data represents mean ± s.e.m of at least 15 independent experiments.
